# Vps501 links sorting nexins to TORC1 regulation in budding yeast

**DOI:** 10.1080/27694127.2022.2111151

**Published:** 2022-10-12

**Authors:** Jacob H. Brock, Muhammad A. Rahman, Richard J. Chi

**Affiliations:** Department of Biological Sciences, University of North Carolina, Charlotte, USA 28223

**Keywords:** Autophagy, SEA complex, sorting nexins SNX-BAR, TORC1

## Abstract

The lysosome in animal cells or the vacuole in plant and yeast cells serve as storage and/or recycling depots and their delimiting membranes host signaling events critical for starvation-induced cellular self-eating or macroautophagy/autophagy. Initiation of autophagy occurs when the Atg1 complex is formed and this process requires the inactivation of the target of rapamycin complex 1 (TORC1), a master regulator of cell growth and metabolism in all eukaryotes. In budding yeast, TORC1 localizes to two independent pools around the vacuolar membrane and in signaling endosomes juxtaposed to the vacuole. These pools are thought to exist independently of nutrient availability and target unique substrates using regulatory elements that are not well understood. One key upstream regulator of TORC1 is the SEA complex (GATOR complex in humans). The SEA complex is a multimeric eight-protein complex (Iml1/Sea1, Rtc1/Sea2, Mtc5/Sea3, Sea4, Seh1, Sec13, Npr2, Npr3) with both inhibiting and activating TORC1 domains, that resides on the vacuolar membrane. However, despite the discovery of the SEA complex over a decade ago, it is still not well understood. In our recent manuscript, we identified a genetic and functional connection between a novel yeast sorting nexin, Vps501, and the SEA complex.

**Abbreviations:**

ORF: open reading frame; PX: Phox homology; SEACIT: sea complex inhibitory domain; SNX: Sorting nexin; TORC1: target of rapamycin complex 1

The lysosome in animal cells or the vacuole in plant and yeast cells serve as storage and/or recycling depots and their delimiting membranes host signaling events critical for starvation-induced cellular self-eating or macroautophagy/autophagy. Initiation of autophagy occurs when the Atg1 complex is formed and this process requires the inactivation of the target of rapamycin complex 1 (TORC1), a master regulator of cell growth and metabolism in all eukaryotes. In budding yeast, TORC1 localizes to two independent pools around the vacuolar membrane and in signaling endosomes juxtaposed to the vacuole. These pools are thought to exist independently of nutrient availability and target unique substrates using regulatory elements that are not well understood. One key upstream regulator of TORC1 is the SEA complex (GATOR complex in humans). The SEA complex is a multimeric eight-protein complex (Iml1/Sea1, Rtc1/Sea2, Mtc5/Sea3, Sea4, Seh1, Sec13, Npr2, Npr3) with both inhibiting and activating TORC1 domains, that resides on the vacuolar membrane. However, despite the discovery of the SEA complex over a decade ago, it is still not well understood. In our recent manuscript [1], we identified a genetic and functional connection between a novel yeast sorting nexin, Vps501, and the SEA complex.

Recently, the sorting nexins (SNX), a diverse family of molecules that play several cellular roles, have been implicated in autophagy. In particular, a subclass of sorting nexins residing on the pre-vacuolar endosome, the SNX-BAR proteins, are of great interest. Interestingly, ORF Ykr078w was annotated as a SNX-BAR in the *Saccharomyces* Genome Database but had no known function. We conducted a phylogenetic analysis and found it to clade with the Vps5 family of proteins and named the gene *VPS501*. To gain insights into Vps501 function, we used a co-immunoprecipitation mass spectrometry approach, and discovered Vps501 interacts with Iml1/Sea1 and Seh1, two subunits of the SEA complex and Kog1 a subunit of TORC1. Additionally, GFP-Vps501 colocalizes with each at the vacuolar membrane.

Because autophagy is the common link between our top proteomic hits, we hypothesized Vps501 also operates in this pathway. To assess bulk autophagy flux, we monitored GFP-Atg8 during nitrogen starvation or rapamycin treatment. We found cells ablated for Vps501 display no defects in starvation-induced or rapamycin-induced autophagy, unless combined with deletions of SEACIT genes, encoding the inhibitory subcomplex of the SEA complex. In particular, *iml1/sea1*Δ *vps501*Δ cells demonstrate a >3-fold loss of autophagic flux as compared to their single mutations, indicating that Vps501 function is genetically associated with Iml1/Sea1 either in parallel or in redundant roles to regulate TORC1 signaling. Additionally, Npr2 and Npr3 are severely destabilized from the vacuolar membrane in *iml1/sea1*Δ *vps501*Δ cells indicating Iml1/Sea1 and Vps501 work synergistically to localize the SEACIT complex to the vacuolar membrane. As others have previously reported, the SEACIT functions as an upstream GTPase activator to inhibit TORC1. Therefore, we hypothesized our autophagic phenotypes are likely due to a broader misregulation of TORC1.

To test for this, we examined the two distinct pools of TORC1 using Kog1-GFP, a subunit of TORC1. In wild-type cells or singly mutated cells, Kog1-GFP localizes around the vacuolar membrane, and to signaling endosomes. However, in *iml1/sea1Δ vps501Δ* cells, Kog1-GFP is significantly reduced on the vacuolar membrane and enriched on signaling endosomes, indicating both pools of TORC1 are aberrant. Recently, Atg13 phosphorylation was found to be a specific target of TORC1 at the signaling endosome. Indeed, when we examined the Atg13 protein levels in *iml1/sea1*Δ *vps501*Δ cells, we found Atg13 is hyperphosphorylated during autophagy induction by nitrogen starvation and rapamycin treatment. This was particularly interesting to us because rapamycin had been shown to directly inhibit TORC1, whereas nitrogen starvation induces TORC1 inactivation via the SEA complex (Figure 1). Therefore, Atg13 hyperphosphorylation in *iml1/sea1*Δ *vps501*Δ cells during rapamycin treatment demonstrates a broad TORC1 signaling defect.

**Figure 1.**
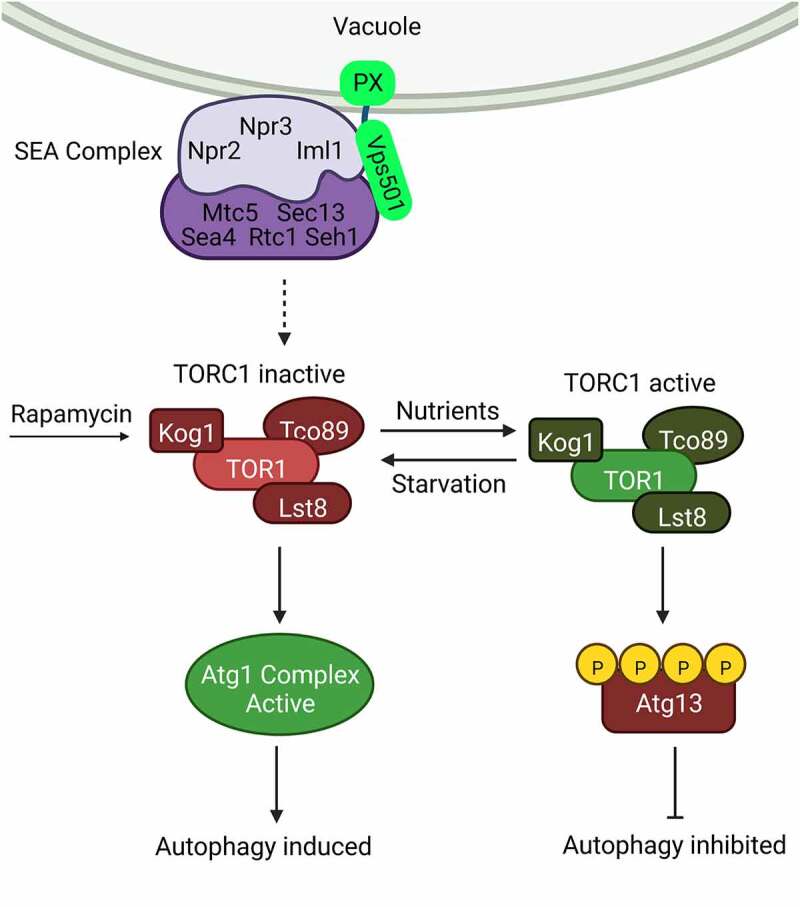
Vps501 is a new link between the SEA complex and TORC1 in budding yeast. Under starvation or rapamycin treatment, Vps501 associates with the SEA complex at the vacuolar membrane using a non-canonical PX domain and a direct interaction with the SEA complex. This interaction promotes SEA complex-mediated inactivation of TORC1 at the vacuole, resulting in the induction of autophagy. In our study, we find the SEA complex and TORC1 are mislocalized in mutant cells resulting in aberrant Atg13 phosphorylation and the inactivation of autophagy. Created in BioRender.com.

Next, we sought to determine how Vps501 is recruited to the vacuolar membrane and the necessity for its function. Our approach centered on three possibilities; 1) Vps501 requires a direct interaction with the SEA complex, 2) Vps501 requires a Phox (PX) homology domain, or 3) a combination of both. First, we tested whether GFP-Vps501 was mislocalized when SEA complex subunits were ablated and found Iml1/Sea1 ablation shifts a large portion of Vps501 to the cytosol with only 30% remaining on the vacuolar membrane, indicating a direct interaction with Iml1/Sea1 is driving a portion of localization. Next, we examined the PX domain of Vps501, a key feature common in all sorting nexins, and found Vps501 has no canonical phosphatidylinositol-3-phosphate (PtdIns3P)-binding motif and instead identified a secondary lipid binding site in the PX domain. When mutated, the *vps501* PX mutant mislocalizes to the cytosol and when combined with an *iml1/sea1*Δ deletion is nearly undetectable at the vacuolar membrane, indicating a combination of both is required for proper localization. This *vps501* PX mutant also fails to complement *iml1/sea1*Δ *vps501*Δ autophagic flux defects indicating vacuolar localization is required for function. Finally, using liposome sedimentation studies, we further defined the major lipid moieties that drive Vps501 binding to the vacuole. We found PtdIns3P drives Vps501 membrane binding, whereas PtdIns(3,5)P_2_, the major lipid species on the vacuolar membrane, has a regulatory role.

Altogether, our study sheds new insights into the overall regulation of TORC1 by a novel sorting nexin and the SEA complex in budding yeast. Recently, there has been growing evidence to suggest SNXs mediate trafficking events required for autophagy; however, many of these roles have been indirect, contributing to autophagy by mobilizing specific lipid moieties for autophagosome biogenesis or by potentiating autophagosome fusion to the vacuole. In our study, we reported the discovery of a novel sorting nexin, Vps501, that localizes to the vacuolar membrane and directly acts with the SEA complex to regulate TORC1 in budding yeast. While Vps501’s mechanistic roles are still unknown, our data suggest Vps501 may act as a structural stabilizer for the SEA complex, using multiple interactions within the SEA complex and lipid specificity from its non-canonical PX domain to facilitate TORC1 inactivation, thereby inducing autophagy (Figure 1). However, future studies are needed to confirm this model and determine if these results are evolutionarily conserved.

## References

[1] Goyal S, Segarra VA, Nitika, Stecher AM, Truman AW, Reitzel AM, Chi RJ. Vps501, a novel vacuolar SNX-BAR protein cooperates with the SEA complex to regulate TORC1 signaling. Traffic. 2022. Epub 2022/02/01. doi: 10.1111/tra.12833. PubMed PMID: 35098628; PMCID: PMC9305297. PMC9305297 35098628

